# Genomic characterization of therapy-associated polyposis reveals an alkylating mutational signature from prior treatment

**DOI:** 10.1172/JCI201216

**Published:** 2026-08-04

**Authors:** Yashika Parashar, Zsofia Sztupinszki, Aurel György Prósz, Xiaolu Wang, Pratyusha Bala, Shweta Cavale, Chinedu Ukaegbu, Sapna Syngal, Asaf Maoz, Leah Biller, Ramona Lim, Matthew B. Yurgelun, Zoltan Szallasi, Nilay S. Sethi

**Affiliations:** 1 Department of Medical Oncology, Dana-Farber Cancer Institute, Boston, Massachusetts, USA.; 2 Department of Medicine, Harvard Medical School, Boston, Massachusetts, USA.; 3 Danish Cancer Institute, Copenhagen, Denmark.; 4 Computational Health Informatics Program, Boston Children’s Hospital, Boston, Massachusetts, USA.; 5 Broad Institute of Massachusetts Institute of Technology and Harvard University, Cambridge, Massachusetts, USA.

**Keywords:** Gastroenterology, Oncology, Colorectal cancer, Molecular genetics

## Abstract

This study shows that chemotherapy leaves lasting DNA scars in precancerous colon polyps decades after treatment, helping explain a rare late complication in childhood cancer survivors.

**To the Editor:** Therapy-associated polyposis (TAP) is a rare, late complication that develops decades after treatment for childhood and young adult cancer (CYAC), most commonly following exposure to abdominal radiotherapy and alkylating chemotherapy. Long-term survivors of CYAC have substantially increased risks of colorectal neoplasia, with colorectal cancer occurring at nearly 11-fold higher rates than in the general population after selected therapies (1–3). Unlike hereditary polyposis syndromes, which arise from inherited defects in epithelial homeostasis, TAP develops only after exposure to cytotoxic therapy. Whether prior cancer therapy accelerates conventional adenoma formation or instead gives rise to a biologically distinct form of colorectal neoplasia has remained unknown (4). We therefore performed the largest clinicopathological and genomic analysis of TAP to define its biological features.

We identified 29 patients with TAP, defined as the development of ≥10 lifetime gastrointestinal polyps in the absence of a known hereditary polyposis syndrome following treatment for CYAC. Median age at primary cancer diagnosis was 16 years (IQR 13–21), and median latency from cancer treatment to polyposis was 29 years (Figure 1, A–C, Supplemental Figure 1, and Supplemental Table 1; supplemental material available online with this article; https://doi.org/10.1172/JCI201216DS1). Hodgkin lymphoma was the most common primary malignancy (Figure 1A), and among patients with available treatment histories, 78% had received alkylating chemotherapy, including mechlorethamine, procarbazine, dacarbazine, or cyclophosphamide. Secondary malignancies were common, particularly basal cell carcinoma, breast cancer, colorectal cancer, and lung cancer (Figure 1B). TAP was diagnosed at a median age of 49 years, substantially earlier than reported cohorts of colonic polyposis of unknown etiology, despite a nearly 3-decade latency from cancer therapy. Patients underwent a mean of 5.4 surveillance colonoscopies, underscoring the substantial long-term clinical burden of TAP. Histopathologic evaluation demonstrated mixed polyp histology with a predominance of adenomas (Figure 1C).

The combination of mixed polyp histology and absence of known hereditary predisposition raised the possibility that TAP might overlap with hereditary mixed polyposis syndrome (HMPS), one of the few hereditary syndromes characterized by mixed adenomatous polyposis. Whole-genome sequencing of TAP adenomas together with HMPS and familial adenomatous polyposis (FAP) controls demonstrated the characteristic 40 kb *GREM1* duplication (5) in every HMPS sample but none in the TAP adenomas (Figure 1D and Supplemental Table 1). Likewise, *GREM1* expression was not consistently increased in TAP (Supplemental Figure 2), excluding HMPS as an explanation for this cohort. These findings support the conclusion that TAP is molecularly distinct from established hereditary polyposis syndromes.

Comparative genomic analyses demonstrated that TAP adenomas harbored substantially greater structural genomic disruption than FAP adenomas, including increased numbers of large deletions, duplications, and genome fragmentation (Figure 1E). Mutational signature analysis identified the expected age-associated signatures SBS1 and SBS5 across all samples. Most notably, SBS25 was robustly detected in TAP adenomas, including specimens lacking matched germline controls, whereas the platinum-associated signature SBS31 remained minimal (Figure 1, F and G). Unlike age-associated signatures, SBS25 has rarely been reported in colorectal neoplasia but has recently emerged as a marker of exposure to methylhydrazine alkylating agents, including procarbazine and dacarbazine (6), both commonly administered during treatment of Hodgkin lymphoma. Its reproducible detection across TAP adenomas decades after treatment demonstrates that prior chemotherapy can leave a durable mutational imprint within premalignant colorectal lesions, providing a molecular record of prior therapeutic exposure. These findings suggest that the genomic consequences of cytotoxic therapy persist long after treatment and remain detectable during premalignant stages of colorectal neoplasia.

To determine whether these genomic alterations were accompanied by changes in epithelial cell state, we established paired normal and adenoma organoids from a patient with TAP. Adenoma-derived organoids demonstrated sustained growth in R-spondin–independent conditions and impaired differentiation compared with matched normal organoids, accompanied by increased SOX9 and reduced KRT20 expression (Figure 1H). These findings parallel features previously described in FAP adenomas (7) and establish TAP organoids as an experimentally tractable platform for investigating how therapy-associated genomic alterations influence epithelial differentiation and adenoma biology.

Together, these findings define TAP as a distinct syndrome characterized by prolonged latency, predominantly adenomatous polyposis, increased structural genomic disruption, and persistent chemotherapy-associated mutational scars. To our knowledge, this represents the first demonstration that a therapy-associated mutational signature can be identified in premalignant colorectal lesions arising decades after cancer treatment. While larger cohorts and mechanistic studies are required to determine how these genomic alterations contribute to adenoma initiation and progression, our findings provide a molecular framework for TAP and support tailored gastrointestinal surveillance strategies for survivors of childhood and young adult cancers.

Study limitations include the modest cohort size, incomplete availability of matched normal tissue, variable sequencing depth, and the retrospective nature of clinical data collection.

For detailed methods, information regarding sex as a biological variable, statistics, study approval, data availability, and author contributions, see the supplemental materials.

## Conflict of interest

NSS is on the scientific advisory board for Astrin Biosciences and Debbie’s Dream Foundation; has consulted for Dewpoint Therapeutics, Bayer, and Department of Gastroenterology at the First Affiliated Hospital of Sun Yat-sen University; receives research funding from Novartis; and is an inventor on patent application PCT 63/208,313 and a second patent application in preparation.

## Funding support

This work is the result of NIH funding, in whole or in part, and is subject to the NIH Public Access Policy. Through acceptance of this federal funding, the NIH has been given a right to make the work publicly available in PubMed Central.

- Virtual Scholar Award from the Department of Defense (CA201084) to NSS.
- NIH grant 1R01CA292507 to NSS.
- Jimmy Fund Walk (Opiela Family) (to the Sethi Lab).
- Craig Baskin (to the Sethi Lab).
- Dave and Carol Fischer (to the Sethi Lab).
- Howard and Wendy Cox (to the Sethi Lab).
- ACS Research Scholar Grant (RSG-24-1321463).
- Discovery Boost Grant (DBG-25-1500423).

## Supplementary Material

Supplemental data

Unedited blot and gel images

Supporting data values

## Figures and Tables

**Figure 1:**
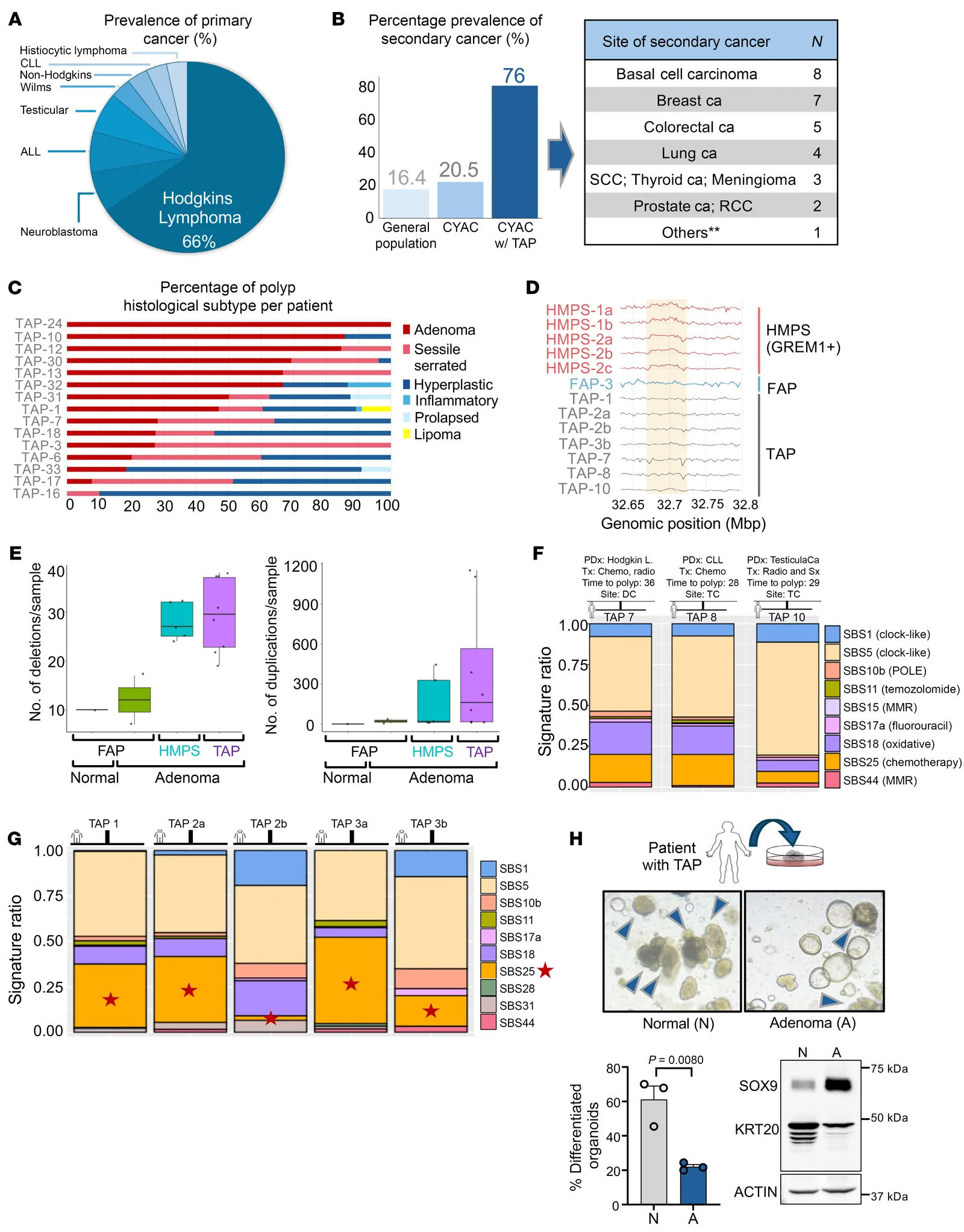
Clinicopathological and genomic analysis of patients with TAP. (**A**) Primary cancer diagnoses in patients with TAP (*n* = 29). (**B**) Secondary cancer prevalence in the general population (>2 million survivors), CYAC survivors (*n* = 14,359), and patients with TAP (*n* = 29). **Melanoma; cholangiocarcinoma; AML; DLBCL; esophageal Ca; vocal cord SCC; osteochondroma; acoustic neuroma; mandibular schwannoma. (**C**) Distribution of polyp histologic subtypes in TAP (*n* = 15). (**D**) Copy number profiles across the *GREM1* locus in HMPS, FAP, and TAP adenomas. (**E**) Structural variant burden in normal tissue and adenomas from FAP and TAP. The box-and-whisker plots depict the minimum and maximum values (whiskers), the upper and lower quartiles, and the median. (**F** and **G**) Single-base substitution (SBS) mutational signature analysis of TAP adenomas with (**F**) and without (**G**) matched germline controls. (**H**) Paired normal and TAP adenoma organoids showing morphology, differentiation, and expression of SOX9 and KRT20. Arrowheads represent organoids that are differentiating in normal and adenoma culture from a patient with TAP. Student’s *t* test. Error bars depict mean with SEM. PDx, primary diagnosis; Tx, treatment; Chemo, chemotherapy; Radio, radiotherapy; Sx, surgery; TC, transverse colon; DC, descending colon; CLL, chronic lymphocytic leukemia; RCC, renal cell carcinoma; SCC, squamous cell carcinoma.

## Version 1 08/04/2026

In-Press Preview

## Version 2 10/01/2026

Electronic publication
