# Supplementary material for: Genomic characterization of therapy-associated polyposis reveals an alkylating mutational signature from prior treatment
Source: J Clin Invest. 2026 Aug 4;136(19):e201216. doi: 10.1172/JCI201216 (PMC13626825; doi:10.1172/JCI201216)
Supplement: Supplementary file 2 [file jci-136-201216-s148.pdf]

Uncropped WB

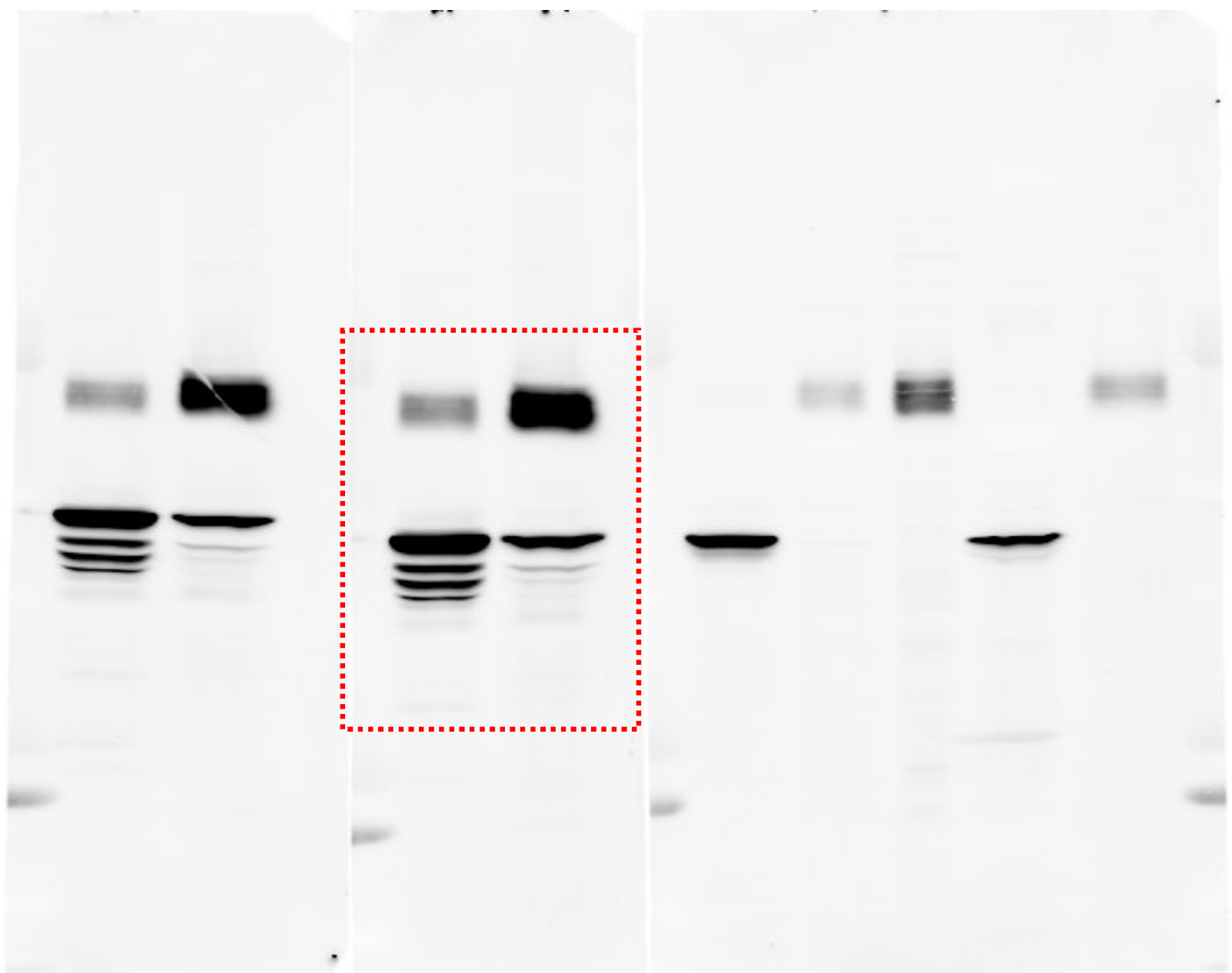

SOX9
KRT20

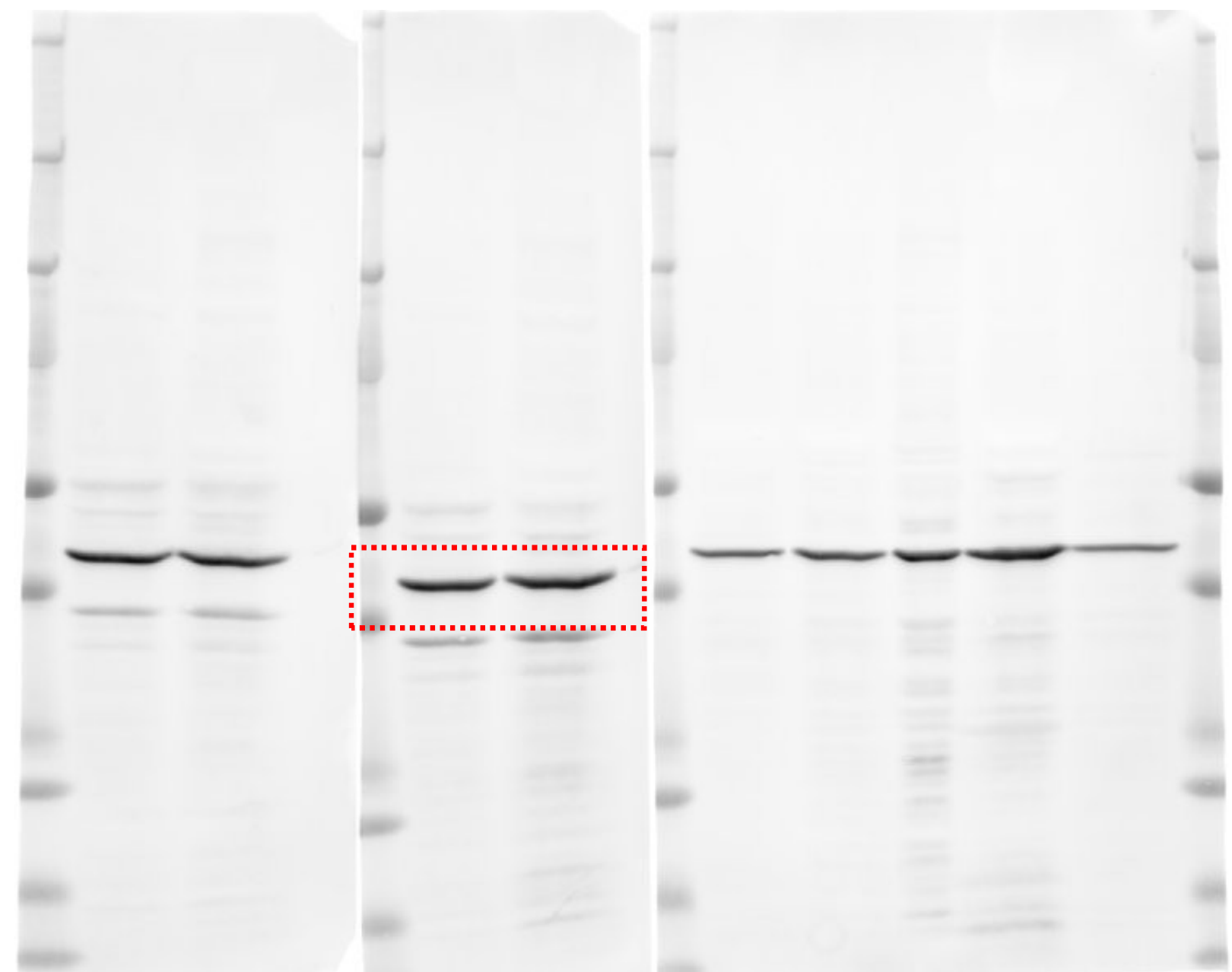

ACTB
